# Supplementary material for: How Quickly Do Proteins Fold and Unfold, and What Structural Parameters Correlate with These Values?
Source: Biomolecules. 2020 Jan 29;10(2):197. doi: 10.3390/biom10020197 (PMC7072309; doi:10.3390/biom10020197)
Supplement: Supplementary file 1 [file biomolecules-10-00197-s001.zip › Supp_TableS2.doc]

**Table 2.** Average values of different parameters for four structural classes of proteins (α, β, α/β and α+β).

| **Average value** | **All (two+multi)** | | | | **Two-state** | | | | **Multi-state** | | | |
| --- | --- | --- | --- | --- | --- | --- | --- | --- | --- | --- | --- | --- |
|  | **α** | **β** | **α/β** | **α+β** | **α** | **β** | **α/β** | **α+β** | **α** | **β** | **α/β** | **α+β** |
| Number of proteins | 31 | 37 | 12 | 28 | 23 | 25 | 1 | 21 | 8 | 12 | 11 | 7 |
| <ln(*kf*)> | 8.49±0.64 | 3.42±0.63 | -0.02±0.85 | 4.71±0.53 | 9.24±0.70 | 4.27±0.80 | 2.8 | 4.95±0.69 | 6.33±1.18 | 1.64±0.82 | -0.27±0.88 | 3.99±0.34 |
| <ln(*ku*)> | 2.03±1.03 | -4.51±1.12 | -8.34±1.64 | -4.76±0.97 | 2.27±1.29 | -2.71±1.35 | -2.7 | -4.17±1.18 | 1.34±1.65 | -8.27±1.60 | -8.85±1.70 | -6.51±1.52 |
| <L> | 80±9 | 92±7 | 168±14 | 90±6 | 77±11 | 74±7 | 168 | 79±5 | 88±12 | 128±13 | 168±15 | 122±11 |
| <Vasa/Sasa> | 3.08±0.07 | 3.27±0.07 | 3.91±0.08 | 3.33±0.06 | 3.00±0.08 | 3.14±0.08 | 4.30 | 3.25±0.07 | 3.28±0.12 | 3.54±0.09 | 3.87±0.08 | 3.59±0.07 |
| <ln(AbsCO)> | 6.60±0.08 | 7.19±0.07 | 7.45±0.07 | 7.08±0.08 | 6.56±0.10 | 7.08±0.08 | 7.39 | 7.09±0.10 | 6.74±0.09 | 7.43±0.08 | 7.45±0.08 | 7.06±0.13 |
| <Rg> | 12.6±0.6 | 12.4±0.3 | 15.0±0.4 | 12.8±0.3 | 12.4±0.7 | 11.5±0.3 | 14.6 | 12.2±0.3 | 13.0±0.9 | 14.2±0.4 | 15.1±0.5 | 14.4±0.6 |
